# Methodology: non-invasive monitoring system based on standing wave ratio for detecting water content variations in plants

**DOI:** 10.1186/s13007-021-00757-y

**Published:** 2021-05-29

**Authors:** Yunjeong Yang, Ji Eun Kim, Hak Jin Song, Eun Bin Lee, Yong-Keun Choi, Jeong Wook Jo, Hyeon Jin Jeon, Ho Hyun Kim, Kwang Jin Kim, Hyung Joo Kim

**Affiliations:** 1 Department of Biological Engineering, Konkuk University, Seoul, 05029 Republic of Korea; 2 Department of Integrated Environmental Systems, Pyeongtaek University, Pyeongtaek, 17869 Republic of Korea; 3 Urban Agriculture Research Division, National Institute of Horticultural and Herbal Science, Chungjoo, 54875 Republic of Korea

**Keywords:** Plant water content, Plant activity monitoring, Standing wave ratio (SWR), *Radermachera sinica*, Coil probe

## Abstract

**Background:**

Water content variation during plant growth is one of the most important monitoring parameters in plant studies. Conventional parameters (such as dry weight) are unreliable; thus, the development of rapid, accurate methods that will allow the monitoring of water content variation in live plants is necessary. In this study, we aimed to develop a non-invasive, radiofrequency-based monitoring system to rapidly and accurately detect water content variation in live plants. The changes in standing wave ratio (SWR) caused by the presence of stem water and magnetic particles in the stem water flow were used as the basis of plant monitoring systems.

**Results:**

The SWR of a coil probe was used to develop a non-invasive monitoring system to detect water content variation in live plants. When water was added to the live experimental plants with or without illumination under drought conditions, noticeable SWR changes at various frequencies were observed. When a fixed frequency (1.611 GHz) was applied to a single experimental plant (*Radermachera sinica*), a more comprehensive monitoring, such as water content variation within the plant and the effect of illumination on water content, was achieved.

**Conclusions:**

Our study demonstrated that the SWR of a coil probe could be used as a real-time, non-invasive, non-destructive parameter for detecting water content variation and practical vital activity in live plants. Our non-invasive monitoring method based on SWR may also be applied to various plant studies.

**Supplementary Information:**

The online version contains supplementary material available at 10.1186/s13007-021-00757-y.

## Background

Measuring the internal water content variation of a live plant is important to understand its physiology and metabolism as well as to retrieve useful information regarding water shortage or over-irrigation [1]. Conventional plant irrigation and its scheduling are based on the analysis of soil water content and meteorological data. The oven-drying technique is generally used for measuring soil water content for research purposes due to its accuracy and simplicity. However, soil measurements performed at specific times are not directly related to the actual water flow in the plant [2]. Therefore, measuring the actual water content of the plant xylem could provide more accurate information regarding the effect of water on plant growth and physiology.

Numerous experimental methods are available to determine the actual water content of live plant components (i.e., leaves, stems, xylem, and roots), including dendrography [3], pressure chamber methods [4], infrared image analysis [5], impedance spectrometry [6], and radar systems [7]. Nevertheless, these methods involve complex experimental procedures that require costly instrumentation. Several methods have been developed to measure plant stem water content directly, including heat pulse systems [2, 8], microneedle sensors [1], and the standing wave ratio (SWR) system [9]. However, these approaches require the insertion of a probe sensor system into the plant xylem, which might cause serious disturbance to the water flow conditions and, consequently, provide unreliable results [10].

A previous study [11] showed that traces of paramagnetic metal components in plants (i.e., humus and water) could induce electrical inductance changes in a coil probe surrounding a plant and also demonstrated that there are clear electrical inductance differences between a live and a dead plant with or without the addition of water. These results suggested that differences in the inductance changes are closely related to differences between the metabolic and physiological activities of live and dead plants. Thus, the non-invasive measurement of stem water flow might be possible using an electromagnetic method.

SWR is estimated by measuring the impedance matching of loads to the characteristic impedance of an antenna, transmission line, or waveguide, and measurements are obtained using a network analyzer [12]. SWR is widely used to estimate radio antenna performance (i.e., resonance point and transmission efficiency) at a specific radio frequency (RF). Due to water in live plant components and their electrical impedance, several studies have shown the transmission of specific RF [13]. Besides, the variation in the water content of a plant has been estimated from SWR values using invasive [9, 14, 15] or non-invasive ring-type sensors [16]. However, these approaches focused only on the water flow in a plant. Therefore, it is necessary to develop a rapid and simple method that will allow the effective estimation of water content variation in plants while observing their vital activity.

In this study, we applied an SWR method for monitoring water content variations and vital activity in plants. Our approach, based on SWR measurements, may represent a simple, rapid, and unique monitoring method for detecting water content variation and vital activities in plants based on SWR.

## Results and discussion

### Changes in SWR for control and experimental vessels under different conditions

**Fig. 1:**
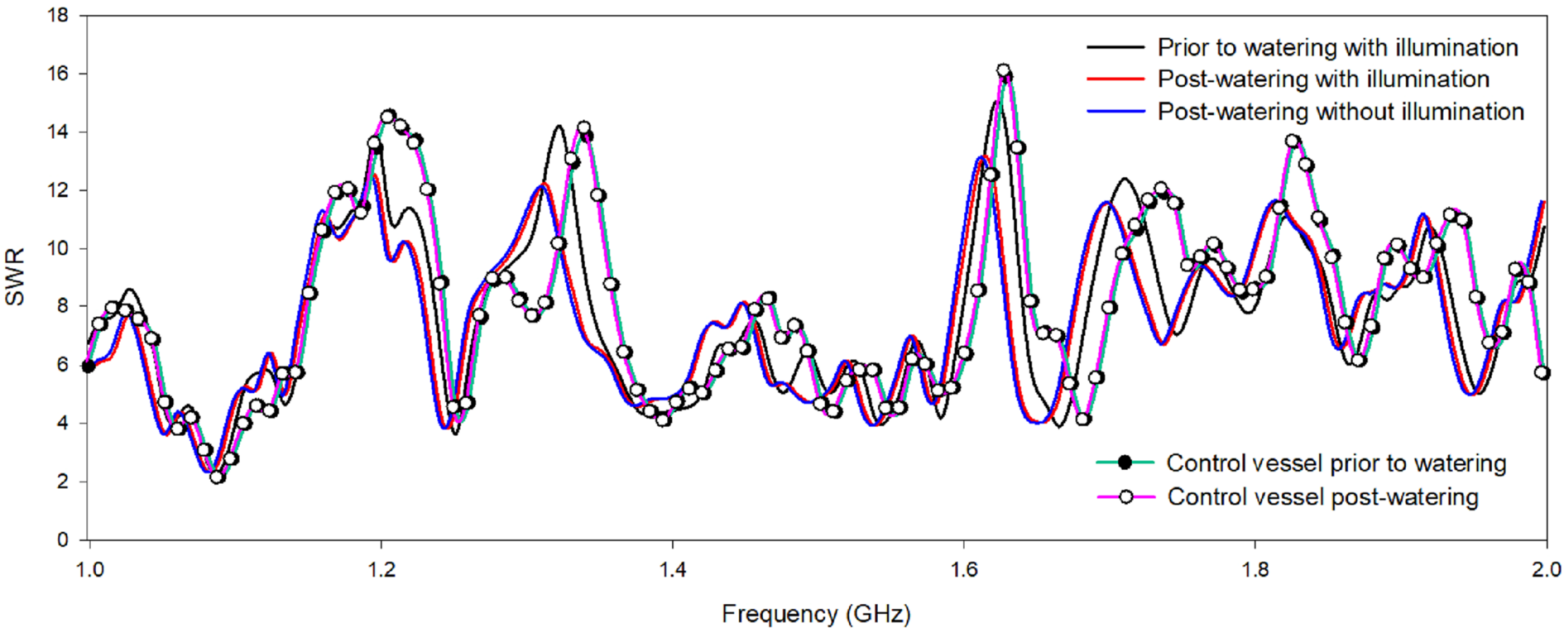
Standing wave ratio (SWR) changes for the experimental plant *Fatoua villosa*. SWR measurement of the coil probe was performed using a network analyzer under various experimental conditions, and the frequency was measured at 1.0–2.0 GHz

An experiment was carried out to obtain preliminary SWR measurements for a vessel containing only soil (control vessel) using a coil probe located above the vessel. Using the same system, a vessel containing the plant *Fatoua villosa* (experimental vessel) was also examined. SWR variations at 1.0–2.0 GHz for the control and the experimental vessels under various conditions are shown in Fig. 1. For the control vessel, SWR showed multiple peaks with a minimum at 2.2 and a maximum at 16.3. No significant variation in the SWR was observed with the addition of water to the control vessel. However, when water was added to the experimental vessel, differences in the SWR, compared with that of the control vessel, were observed throughout the frequency range. As shown in Fig. 1, the experimental vessel induced two peak frequencies, and its SWR values were lower than those of the control vessel throughout the frequency range. For instance, the control vessel showed a peak at 1.629 GHz with an SWR of 16.1, whereas the experimental vessel showed a left-shifted peak at 1.620 GHz with an SWR of 14.8 (Fig. 1A). At frequency ranges of 1.197–1.202 GHz (Fig. 1B), 1.323–1.341 GHz (Fig. 1C), 1.710–1.737 GHz (Fig. 1D), and 1.917–1.935 GHz (Fig. 1E), reduced and shifted SWR were observed for the experimental vessel, probably due to the presence of stem water and magnetic particles in the stem water flow that changed the RF characteristics of the coil probe [11].

To evaluate the effect of water addition on *F. villosa*, SWR measurements were obtained after 3 d of no watering and 3 h post-watering. As shown in Fig. 1, watering generated major and minor SWR changes compared with no watering throughout the frequency range. For instance, post-watering SWR changed by 2.4 ± 0.61% at 1.026 GHz, 2.1 ± 0.83% at 1.197 GHz, 3.1 ± 0.91% at 1.467 GHz, and 2.27 ± 0.57% at 1.620 GHz. These results indicated that interaction between water and the live plant induced SWR changes at specific frequencies that the coil probe could monitor. These SWR changes were probably caused by plant physiological activities, including transpiration and water transportation, which affected the RF characteristics of the coil probe [17].

**Fig. 2:**
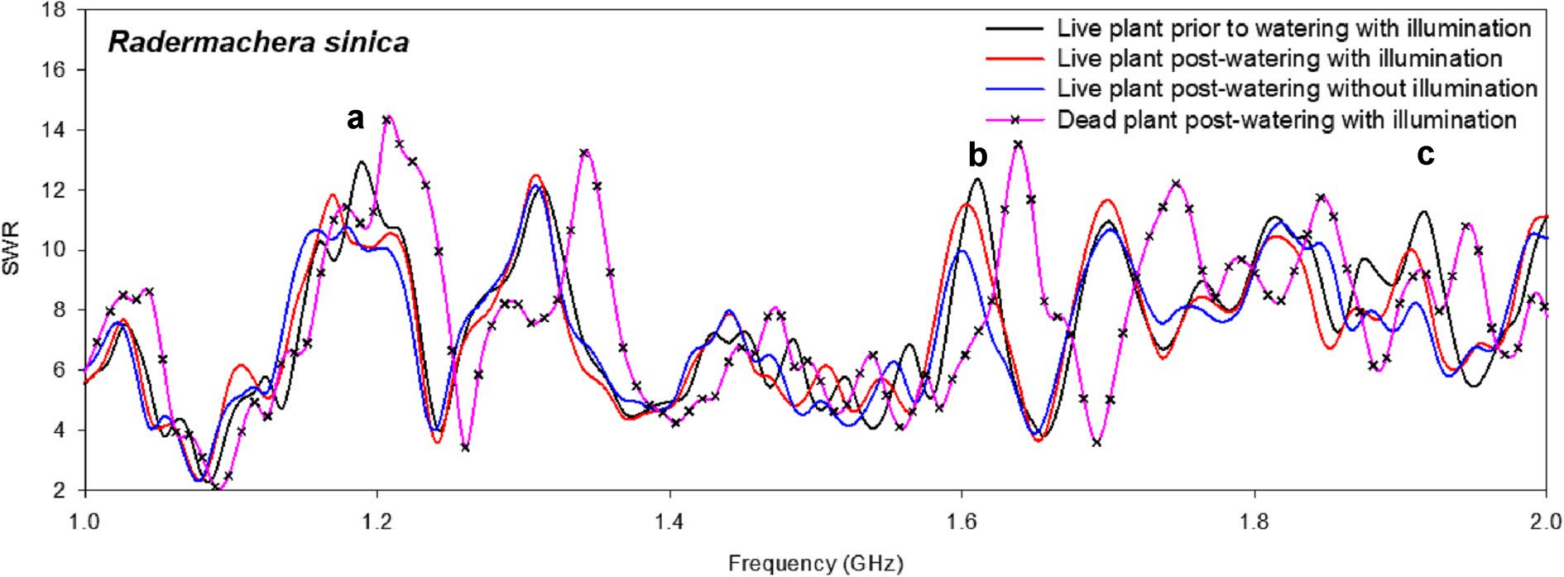
Standing wave ratio (SWR) changes for the experimental plant *Radermachera sinica*. SWR measurement of the coil probe was performed using a network analyzer under various experimental conditions, and the frequency was measured at 1.0–2.0 GHz

To evaluate the effect of illumination on *F. villosa*, SWR measurements were obtained after 3 d of no watering and 3 h post-watering without illumination. No differences were observed in the SWR post-watering with or without illumination. However, when *Radermachera sinica* was used as an experimental plant, watering with or without illumination yielded a markedly different SWR at 1.0–2.0 GHz. As shown in Fig. 2, watering under illumination decreased the SWR by 11.8 ± 0.79% at 1.170 GHz (Fig. 2A), increased it by 10.9 ± 0.85% at 1.611 GHz (Fig. 2B), and decreased it by 9.9 ± 0.93% at 1.908 GHz (Fig. 2C). The dead plants showed significantly different SWR compared with those of the live plants but almost identical with those of the control vessel (Fig. 2). Therefore, the plant configuration (i.e., leaf number, leaf shape, and stem length) and physiological activities (i.e., photosynthesis, stem water flow, water content, and concentration of magnetic materials) might affect SWR throughout the frequency range. To evaluate the accuracy and reproducibility of our results, further experiments were carried out with various live plants under the same experimental conditions.

**Fig. 3:**
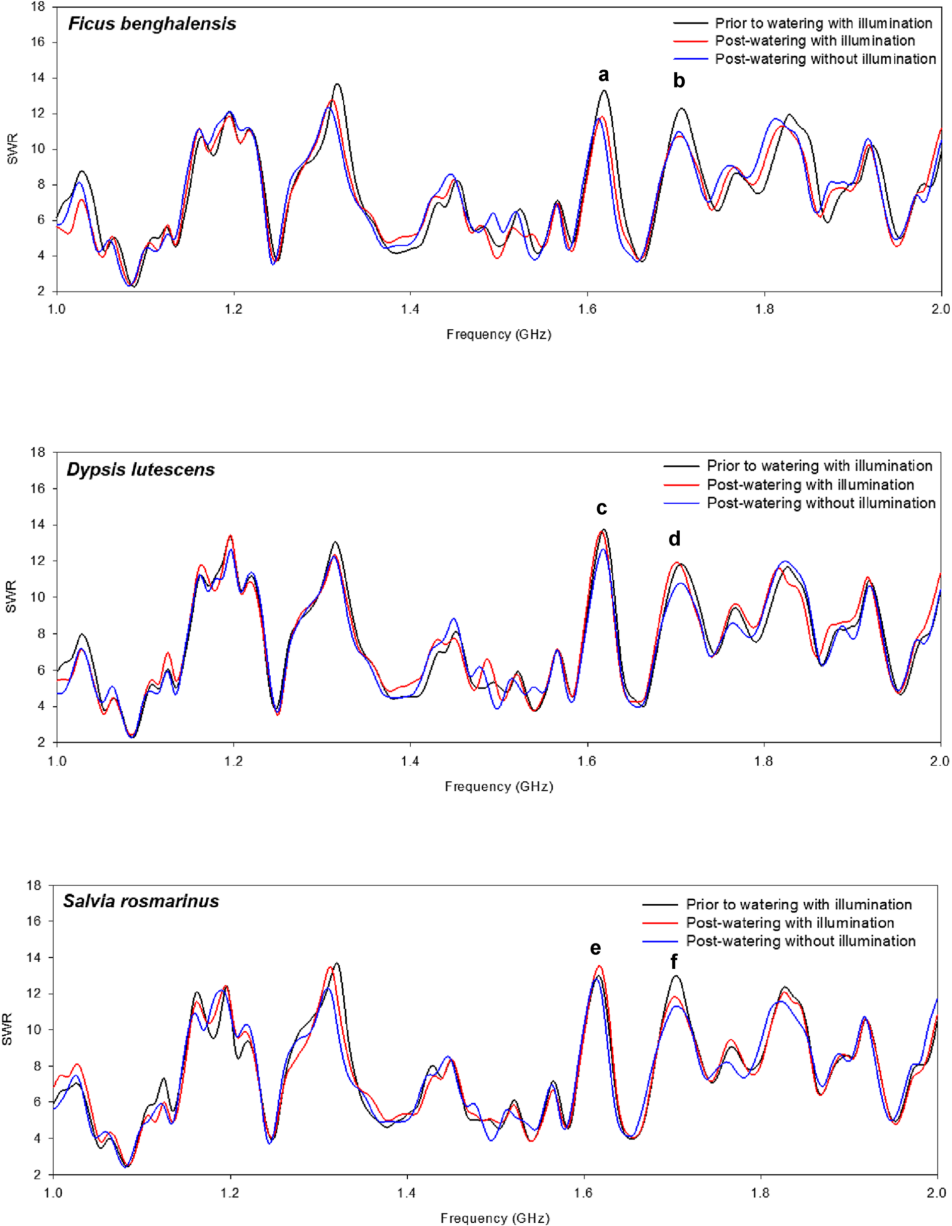
Standing wave ratio (SWR) changes for experimental plants *Ficus benghalensis*, *Dypsis lutescens*, and *Salvia Rosmarinus*. SWR measurement of the coil probe was performed using a network analyzer under various experimental conditions, and the frequency was measured at 1.0–2.0 GHz

Various experimental plants were examined using the same monitoring system after 7 d, 5 d, and 3 d of no watering for *Ficus benghalensis*, *Dypsis lutescens,* and *Salvia rosmarinus*, respectively, as well as at 3 h post-watering, with or without illumination. As shown in Fig. 3, no watering or watering with or without illumination yielded diverse SWR for each plant species. For *F. benghalensis*, watering markedly decreased the SWR at 1.620 GHz and 1.710 GHz (Fig. 3A, B), whereas illumination did not have a significant effect (Fig. 3). For *D. lutescens*, watering without illumination decreased the SWR at 1.620 and 1.702 GHz (Fig. 3C, D). For *S. rosmarinus*, watering with illumination increased the SWR at 1.620 GHz but decreased it with or without illumination at 1.701 GHz (Fig. 3E, F). Variations among the plant species could be attributed to differences in evapotranspiration rates, physiological activities, and water contents.

### Effect of watering and illumination on SWR in R. sinica

**Fig. 4:**
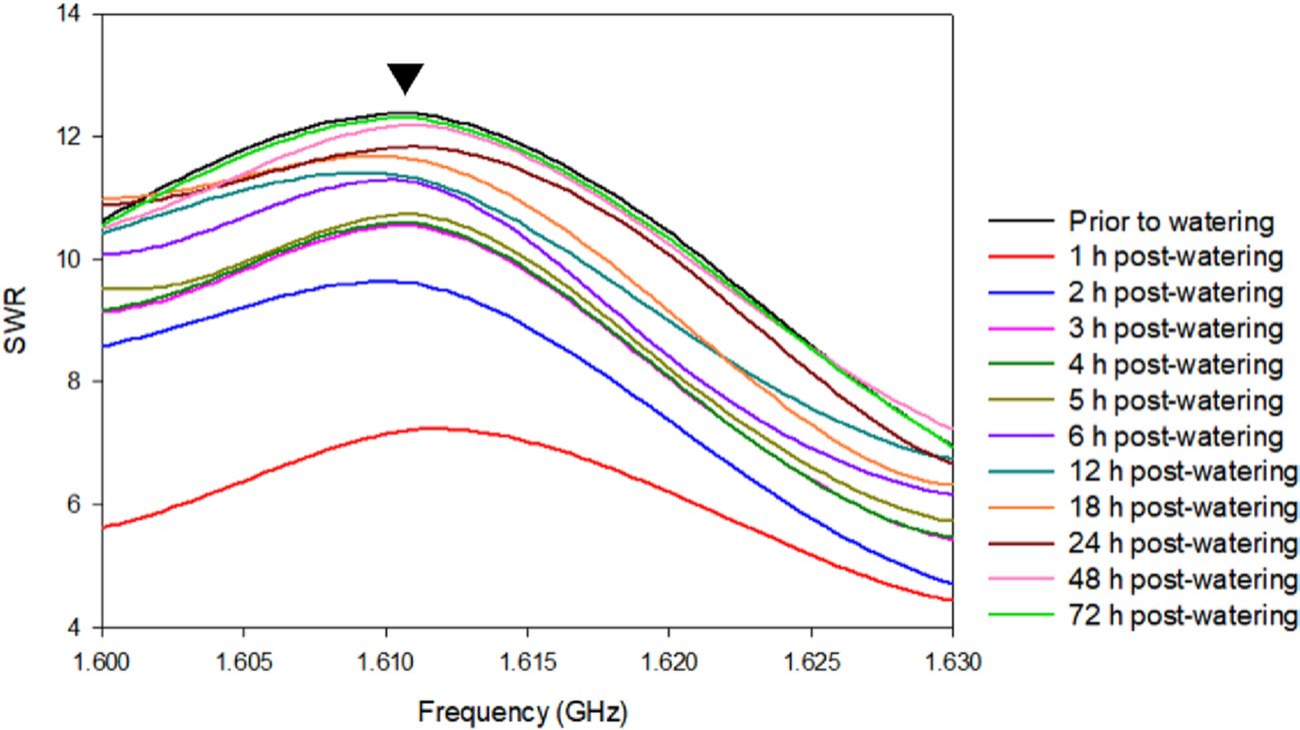
Standing wave ratio (SWR) changes for *Radermachera sinica* prior to and 3–72 h post-watering. A narrow frequency range of 1.600–1.630 GHz was set. The plant remained without water for 7 d prior to the experiment. Triangle indicates the frequency of 1.611 GHz

To study the SWR-based activities post-watering, we selected *R. sinica* as it yielded the highest and more reproducible SWR variation under different watering and illumination conditions at the frequency range of 1.600–1.630 GHz (Fig. 4). We observed a rapid decrease in the SWR at 1 h post-watering, followed by a gradual increase with time and stabilization at 3 h post-watering. Significant error signals (*p* > 0.05) were observed at 1 h post-watering but reduced at 3 h post-watering. The SWR continually decreased up to 72 h post-watering. Repeated experiments showed that 1.611 GHz was the most reproducible frequency for *R. sinica* water content monitoring using SWR measurements (Fig. 4). Thus, the effect of watering at 3-d intervals on live and dead plants with or without illumination was further monitored at 1.611 GHz for 72 d.

**Fig. 5:**
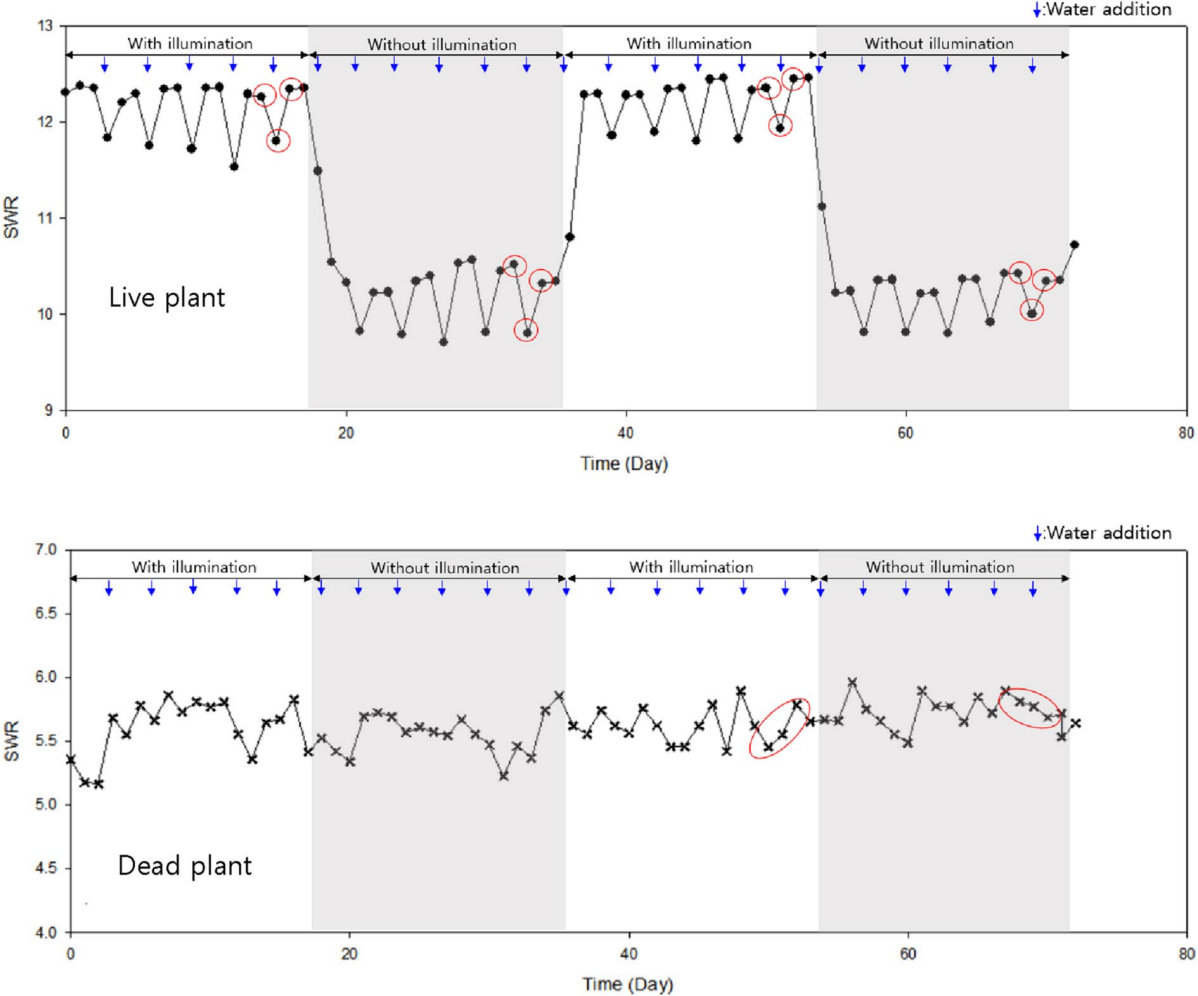
Standing wave ratio (SWR) changes for live and dead *Radermachera sinica* with or without illumination. The frequency was measured at a specific frequency of 1.611 GHz. SWR measurements were also performed 3 h post-watering. Circles indicate the sampling point for obtaining dry weight measurements

As shown in Fig. 5, the effect of plant photosynthetic activity on the SWR of live and dead plants was assessed for 72 d with (0–17 d and 36–53 d) and without illumination (18–35 d and 54–72 d). At 0–17 d, watering rapidly decreased the SWR of the live plants, but the values gradually recovered at 3 d post-watering. However, the live plants showed a higher decrease in the SWR post-watering without illumination compared with that post-watering with illumination. No significant differences were observed in the SWR at 0–17 d and 36–53 d as well as those at 18–35 d and 54–72 d (Fig. 5). These results indicated that photosynthesis was related to the SWR and that the plant vital activities related to photosynthesis could be assessed using this method. Variation in the SWR post-watering was only observed for the live plant.

**Fig. 6:**
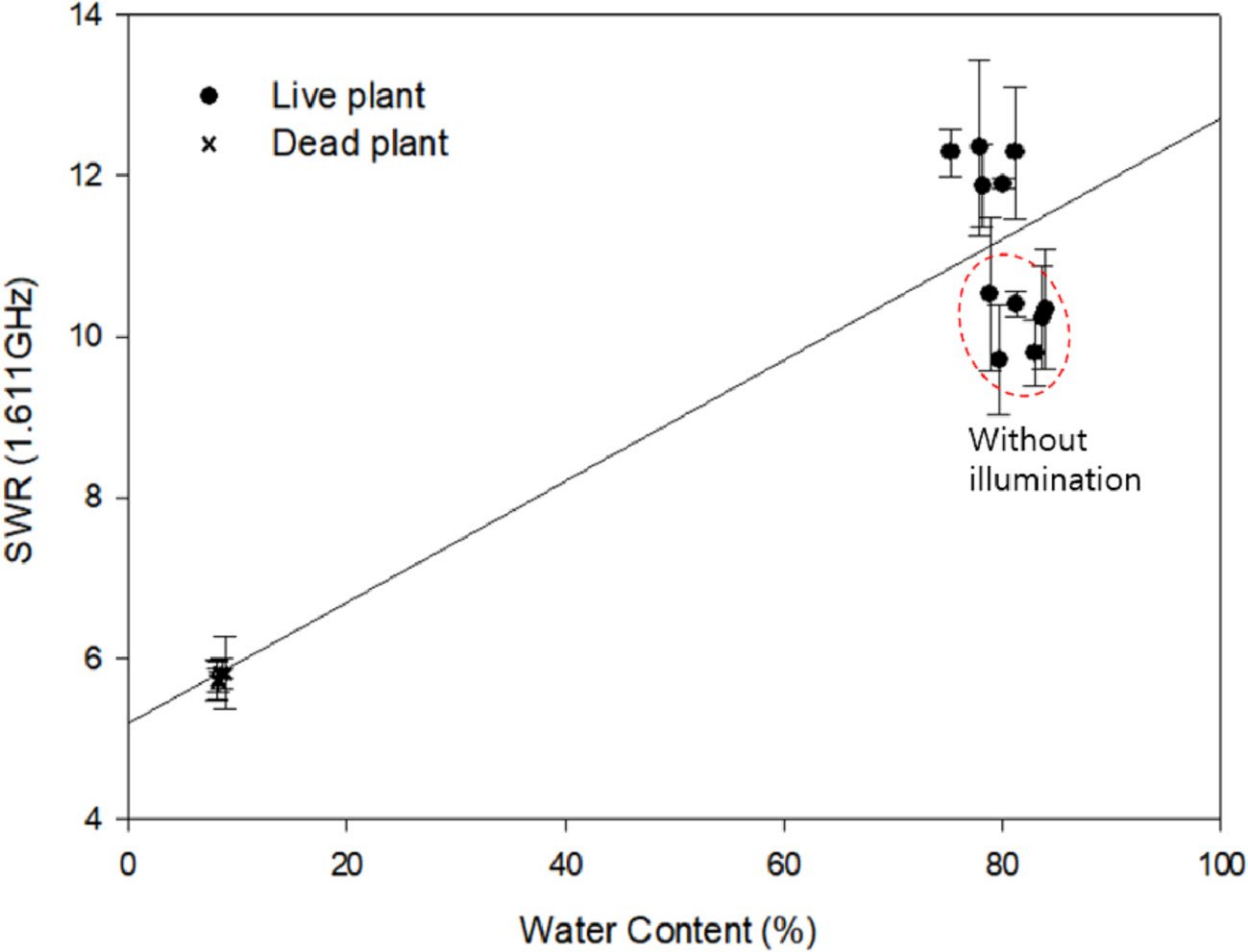
Correlation between standing wave ratio (SWR) and *Radermachera sinica* water content at a specific frequency. The water content was estimated using the dry weight method. Dotted circles indicate the SWR and dry weight of the plant with or without illumination. After plant death, the water content was analyzed at the final stages of the experiment due to limited sample availability. (*r*^2^ = 0.9346)

To identify the correlation between the SWR and the water content, we used the dry weight method. To this end, mainly leaves from the live plants and stems from the dead plants were collected throughout the 72 d, and the SWR at 1.611 GHz was recorded at the sampling time. As shown in Fig. 6, variations in the SWR and water content were relatively high with or without illumination for the live plant, whereas no significant variations in the SWR or the water content were observed for the dead plant. Besides, the live plant without illumination showed slightly higher water content and lower SWR than that with illumination. Previous studies suggested that dark conditions decrease transpiration due to stomatal closing, resulting in increased water content [18] and negatively affecting photosynthesis [19]. Our study showed that depressed photosynthesis due to lack of illumination impaired vital activities such as water flow, transpiration, and evapotranspiration. Variations in the SWR and water content induced changes in the RF characteristics of the coil probe. Therefore, monitoring of SWR changes at a specific frequency range could provide a possible way to assess vital activities in plants.

Previous studies used water monitoring techniques that required invasive sensors and time-consuming procedures. In contrast, our proposed monitoring system is non-invasive and provides accurate results in a relatively short time. It is known that SWR variations based on capacitance or inductance changes [20] caused by mobile paramagnetic components in plant water might allow the non-invasive assessment of stem water content. In the present study, variations in the SWR at a specific frequency range were used to monitor changes in the water content and vital activities of experimental plants. Multiple resonance frequency points with resonance frequency shifts have great potential for developing plant activity scanners.

Preliminary experiments with different plant species [11] indicated that electrical factors, such as the diameter and winding number of the coil probe, significantly affect the measurement results. Higher frequency ranges (i.e., 2.0–4.0 GHz) produced noticeable SWR variation post-watering (Additional file 3), whereas frequencies < 1.0 GHz were not used because of the increased noise (Additional file 4). Our results indicated that electrical (i.e., winding number and diameter of the probe), biological (i.e., plant age, growth, type, and size), and environmental (i.e., water, illumination, and fertilization) factors could affect the SWR. Therefore, our monitoring system could be used for other plant species and different conditions using modified probes.

## Conclusions

The aim of the present study was to establish a simple, almost real-time, non-invasive system for monitoring the water content and vital activities in plants. Using a simple coil probe, we demonstrated that SWR variations might allow detecting changes in the water content or vital activities of plants at a fixed frequency. However, more research is needed to improve the coil type probe dimensions and verify the effectiveness and overall applicability of our monitoring system in various plant species, plant physiological conditions, sample sizes, and experimental conditions.

## Methods

### Plant preparation

In this study, we examined the effect of water addition and illumination on five different experimental plants: *F. villosa* (height: approximately 550 mm)*, R. sinica* (height: approximately 700 mm), *F. benghalensis* (height: approximately 500 mm), *D. lutescens* (height: approximately 550 mm), and *S. Rosmarinus* (height: approximately 200 mm) using the SWR (Additional file 1). The selection criteria of the experimental plants were their easy indoor growing characteristics and shape differences [21]. All experimental plants were purchased from the Yangjae Flower Market, Seoul, Republic of Korea. The plants were cultivated for a month before the experiment in a vessel (200 × 180 mm diameter) containing 500 g of organic soil (Jeil Co., Seoul, Korea) under illumination with an intensity of 80 μmol/m^2^/s at 22–25 °C. The plants were watered with tap water (200 ml) every 7 d for *F. benghalensis*, 5 d for *D. lutescens*, and 3 d for *R. sinica, S. rosmarinus*, and *F. villosa.*

### SWR-based monitoring system

**Fig. 7:**
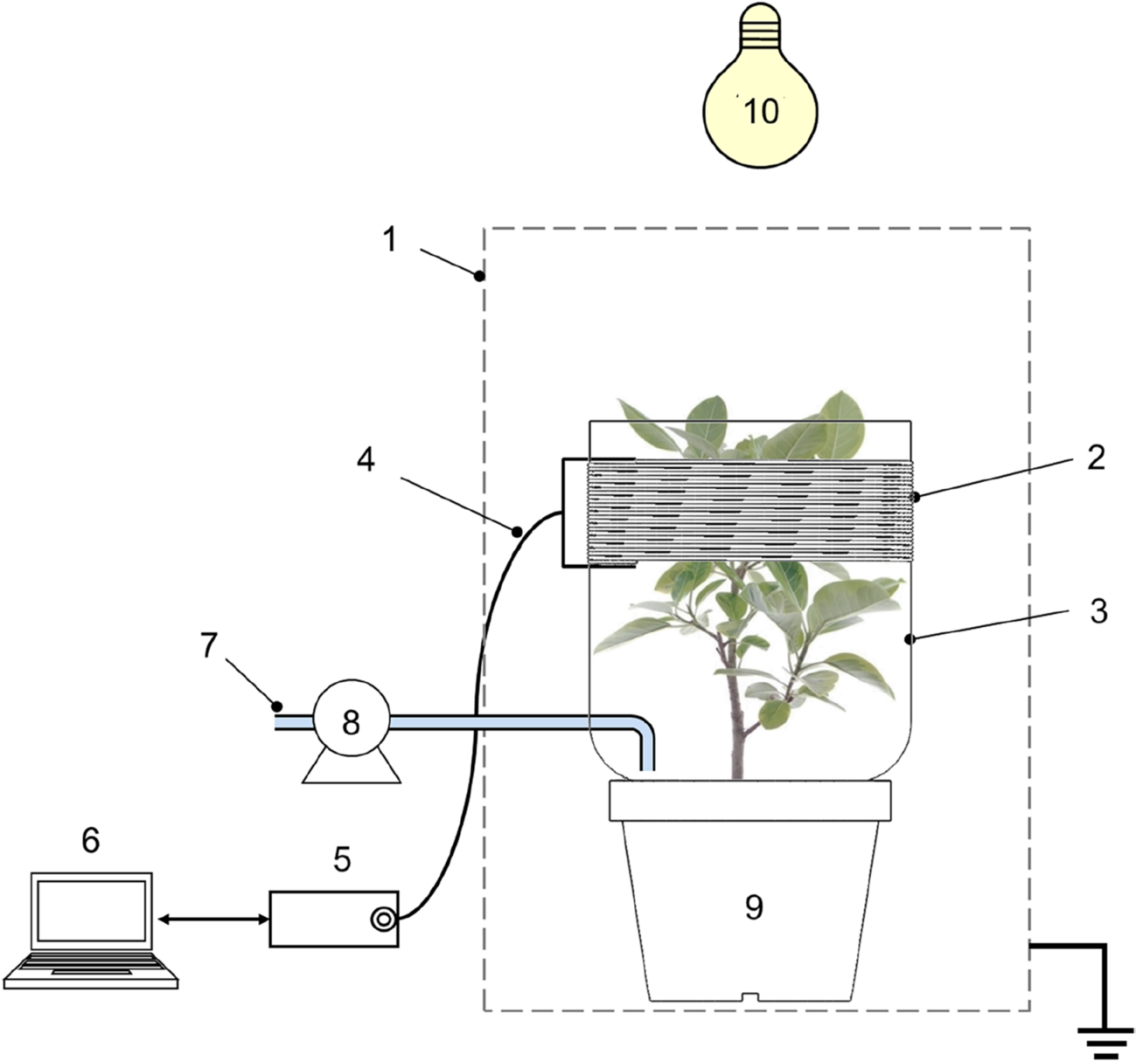
Schematic representation of the monitoring system: (1) Faraday cage, (2) coil probe, (3) supporter, (4) coaxial cable and N-type connectors, (5) network analyzer, (6) PC and control software, (7) tap water, (8) peristaltic pump, (9) experimental plant (*Ficus benghalensis*), and (10) LED light

Figure 7 shows a schematic diagram of the SWR-based monitoring system used in this study. To minimize the effect of external electromagnetic fields, the plant and system were placed inside a grounded Faraday cage (710 × 350 × 1380 mm) covered with a copper mesh (70-gauge, 0.15 mm), and the plant was watered with tap water (200 ml) using a peristaltic pump. Metabolic activity was maintained under illumination (PAR38, Philips, Holland) with an intensity of 110 μmol/m^2^/s at 25 ± 1 °C. The coil probe was constructed using a 0.3 mm copper wire wound 100 times around an acrylic bobbin (250 × 1 mm), and the ends of the wire were tabbed for connection to the SWR meter. The inductance and impedance of the empty coil probe (i.e. without the load of the plant and/or vessel) were 3.481 mH and 35.235 MΩ (at 1.611 GHz), respectively. The inductance and impedance were measured by an inductance meter (LC200A, Brightwin Electronics, China). The experimental plant was then surrounded with the coil probe at a height of 200 mm from the cultivate vessel (Fig. 7, Additional file 2). The tabbed ends of the coil probe were connected to a computer-controlled network analyzer (MS46121B, Anritsu, Japan) using an N-type connector and a coaxial cable (15NNF50-1.5C, Anritsu, Japan). When the system was initially set-up with the plant, frequencies of 1.0–2.0 GHz were scanned, and the SWR variation from the probe was monitored. The SWR results from the probe were recorded using control software (Shockline, Anritsu, Japan). For the control experiment, the SWR was measured with an empty vessel that contained only organic soil (500 g) under the same experimental conditions. For the live plant measurement, the experimental plant was placed in a vessel with organic soil and watered. After 3 h of stabilization, the first SWR measurement was obtained. The measurement for the “before water addition condition” was obtained at 7 d for *F. benghalensis*, 5 d for *Dypsis lutescens*, and 3 days for *R. sinica*, *S. rosmarinus*, and *F. villosa*. In the illumination condition experiment, illumination was turned off for at least 12 h before the water was added. All SWR experiments were performed at least in triplicate, and the average values were plotted.

### Plant death and moisture measurement methods

After the experiment, the live plant was killed using a heat gun (KX-1800, Black and Decker, USA) for 5 min. The dead plant was then left for 30 d under the cultivation conditions described above without the addition of water to ensure complete termination of physiological activity. The dead plant was then used for all the relevant experiments (all plant leaves were deciduous during the treatment). Plant moisture content measurements were conducted based on the dry weight [22]. Leaves from the live plants and stems from the dead plants were used as the moisture measurement samples.

## Supplementary Information

**Additional file 1:** Microsoft Word Document.docx. The experimental plants used in this study.

**Additional file 2:** Microsoft Word Document.docx. A plant sample, vessel, coil probe, the N-type connector and the coaxial cable used in this study.

**Additional file 3:** Microsoft Word Document.docx. Standing wave ratio (SWR) changes for the experimental plant *Radermachera sinica* at 2.0–4.0 GHz.

**Additional file 4:** Microsoft Word Document.docx. Standing wave ratio (SWR) changes for the experimental plant *Radermachera sinica* at 0.0–1.0 GHz.

## Data Availability

All data generated or analyzed during this study are included in this published article (and its additional files).

## Abbreviations

RF: Radio frequency
SWR: Standing wave ratio

## References

[1] Baek S, Jeon E, Park KS, Yeo K-H, Lee J (2018). Monitoring of water transportation in plant stem with microneedle sap flow sensor. J Microelectromech Syst.

[2] González-Altozano P, Pavel EW, Oncins JA, Doltra J, Cohen M, Paco T, Massai R, Castel JR (2008). Comparative assessment of five methods of determining sap flow in peach trees. Agric Water Manag.

[3] Klepper B, Browning VD, Taylor HM (1971). Stem diameter in relation to plant water status. Plant Physiol.

[4] Hellkvist J, Richards GP, Jarvis PG (1974). Vertical gradients of water potential and tissue water relations in Sitka spruce trees measured with the pressure chamber. J Appl Ecol.

[5] Poblete-Echeverría C, Sepulveda-Reyes D, Ortega-Farias S, Zuñiga M, Fuentes S (2016). Plant water stress detection based on aerial and terrestrial infrared thermography: a study case from vineyard and olive orchard. Acta Hortic.

[6] Hamed KB, Zorring W, Hamzaoui AH (2016). Electrical impedance spectroscopy: A tool to investigate the responses of one halophyte to different growth and stress conditions. Comput Electron Agric.

[7] Santos LC, Santos FN, Morais R, Duarte C (2021). Potential non-invasive technique for accessing plant water contents using a radar system. Agronomy.

[8] Bernal AL, Testi L, Villalobos FJ (2001). Using the compensated heat pulse method to monitor trends in stem water content in standing trees. J Hortic Sci Biotech.

[9] Gao C, Zhao Y, Zhao Y (2019). A novel sensor for noninvasive detection of in situ stem water content based on standing wave ratio. J Sensors.

[10] Ruger S, Ehrenberger W, Arend M, GeBner P, Zimmermann G, Zimmermann D, Bentrup FW, Nadler A, Raveh E, Sukhorukov VL, Zimmermann U (2010). Comparative monitoring of temporal and spatial changes in tree water status using the non-invasive leaf patch clamp pressure probe and the pressure bomb. Agric Water Manag.

[11] Yang Y, Lee EB, Kim JE, Song HJ, Choi Y, Kim KJ, Kumaran RS, Lee SH, Yang Y, Kim HH (2019). Monitoring plant moisture content using an induction coil Sensor. Bull Korean Chem Soc.

[12] Patil RV, Xu S, Hoek AN, Rusinko A, Feng Z, May J, Hellberg M, Sharif NA, Wax MB, Irigoyen M, Carr G, Brittain T, Brown P, Colbert D, Kumari S, Baradaraj K, Mitra AK (2016). Rapid identification of novel inhibitors of the human aquaporin-1 water channel. Chem Biol Drug Des.

[13] Jamaludin D, Aziz SA, Ahmad D, Jaafar HZE (2015). Impedance analysis of *Labisia pumila* plant water status. Inf Process Agric.

[14] Wang H, Zhao Y. Measuring water content variations in stems by standing wave ratio principle. In: Proceedings—2010 IEEE conference on mechatronics and automation (ICMA), Xian, China; 2010. p. 124–8. 10.1109/ICMA.2010.5588432.

[15] Manatrinon S, Chantaweesomboon W, Chinrungrueng J, Kaemarungsi K. Moisture sensor based on standing wave ratio for agriculture industry. In: Proceedings—2016 7th international conference of information and communication technology for embedded systems (IC-ICTES), Bangkok, Thailand; 2016. p. 51–6. 10.1109/ICTEmSys.2016.7467121.

[16] Gao C, Zhao Y, Zhao Y (2019). A Novel sensor for noninvasive detection of in situ stem water content based on standing wave ratio. J Sensor.

[17] Vian A, Davies E, Gendraud M, Bonnet P (2016). Plant responses to high frequency electromagnetic fields. Biomed Res Int.

[18] Nakayama FS, Ehrler WL (1964). Beta ray gauging technique for measuring leaf water content changes and moisture status of plants. Plant Physiol.

[19] Zlatev Z (2012). An overview on drought induced changes in plant growth, water relations and photosynthesis. Emir J Food Agric.

[20] Kraus W, Fantz U, Heinemann B, Franzen P (2015). Solid state generator for powerful radio frequency ion sources in neutral beam injection systems. Fusion Eng Des.

[21] Kattenborn T, Fassnacht FE, Schmidtlein S (2018). Differentiating plant functional types using reflectance: which traits make the difference?. Remote Sens Environ.

[22] Jin X, Shi C, Yu CY, Yamada T, Sacks EJ (2017). Determination of leaf water content by visible and near-infrared spectrometry and. multivariate calibration in *Miscanthus*. Front Plant Sci.

