# Supplementary material for: Methodology: non-invasive monitoring system based on standing wave ratio for detecting water content variations in plants
Source: Plant Methods. 2021 May 29;17:56. doi: 10.1186/s13007-021-00757-y (PMC8164761; doi:10.1186/s13007-021-00757-y)
Supplement: Supplementary file 1 — Additional file 1: Microsoft Word Document.docx. The experimental plants used in this study. [file 13007_2021_757_MOESM1_ESM.docx]

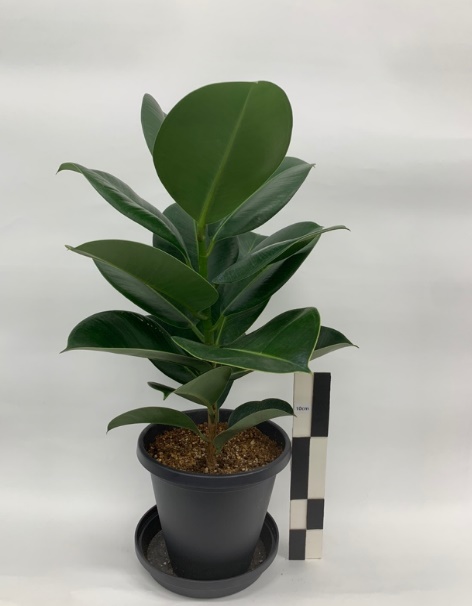

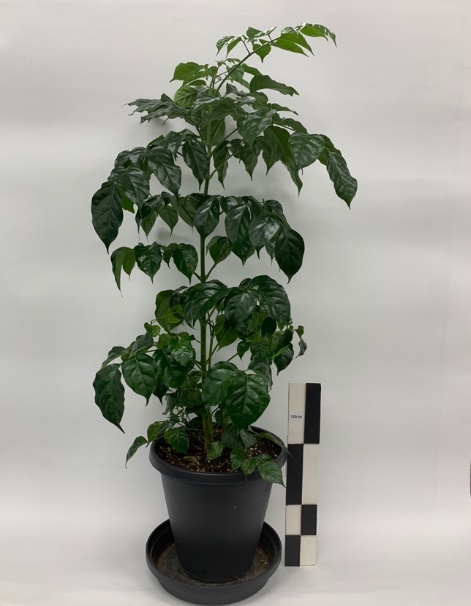

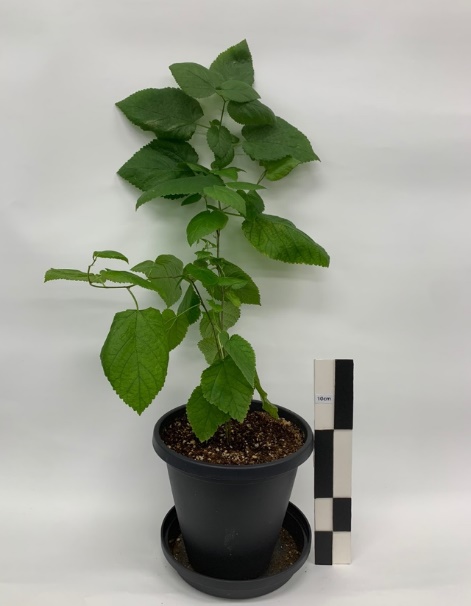


100

100

100

*Fatoua villosa Radermachera sinica Ficus benghalensis*


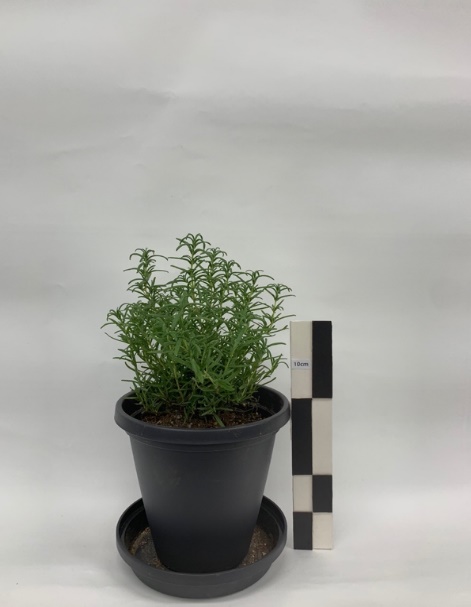
*
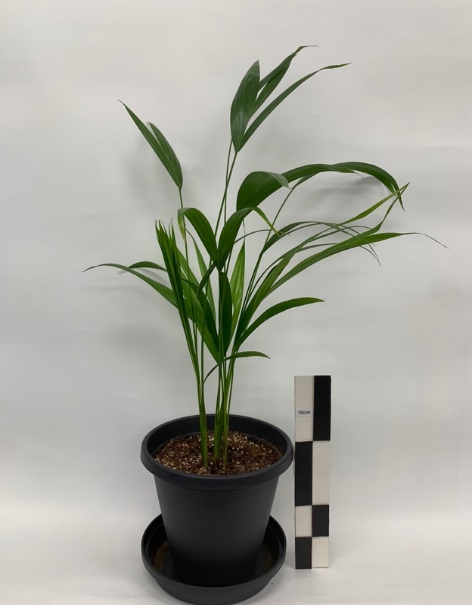
*

100

100

*Dypsis lutescens Salvia rosmarinus*

**Additional file 1** The experimental plants used in this study. The units are in mm.
