# Supplementary material for: Methodology: non-invasive monitoring system based on standing wave ratio for detecting water content variations in plants
Source: Plant Methods. 2021 May 29;17:56. doi: 10.1186/s13007-021-00757-y (PMC8164761; doi:10.1186/s13007-021-00757-y)
Supplement: Supplementary file 2 — Additional file 2: Microsoft Word Document.docx. A plant sample, vessel, coil probe, the N-type connector and the coaxial cable used in this study. [file 13007_2021_757_MOESM2_ESM.docx]

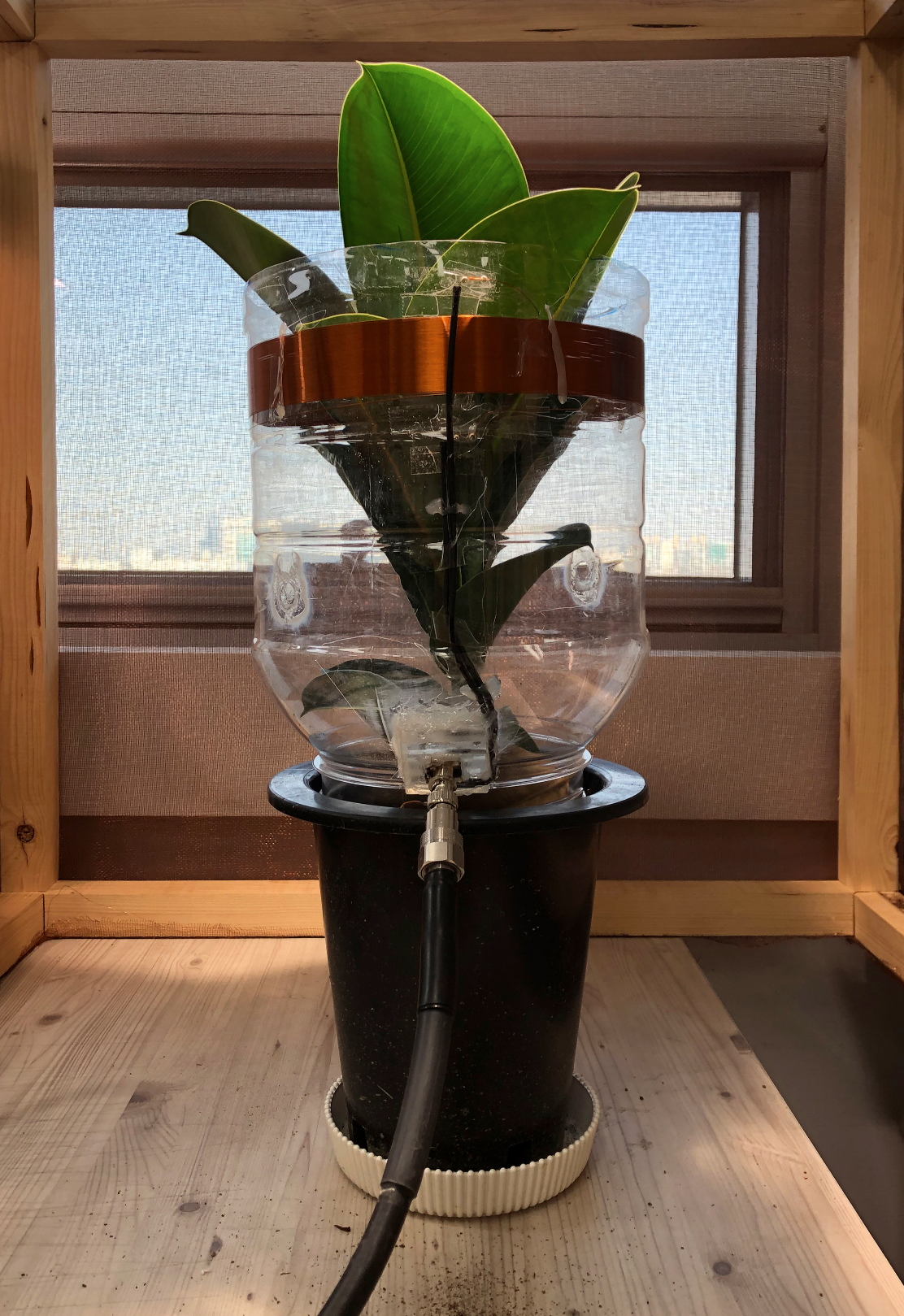


**Plant**

**Coil probe**

**N-type**

**Connector**

**Vessel**

**Coaxial**

**cable**

**Additional file 2** Image of a plant sample, vessel, coil probe, N-type connector, and coaxial cable used in this study.
