# Supplementary material for: Methodology: non-invasive monitoring system based on standing wave ratio for detecting water content variations in plants
Source: Plant Methods. 2021 May 29;17:56. doi: 10.1186/s13007-021-00757-y (PMC8164761; doi:10.1186/s13007-021-00757-y)
Supplement: Supplementary file 4 — Additional file 4: Microsoft Word Document.docx. Standing wave ratio (SWR) changes for the experimental plant Radermachera sinica at 0.0–1.0 GHz. [file 13007_2021_757_MOESM4_ESM.docx]

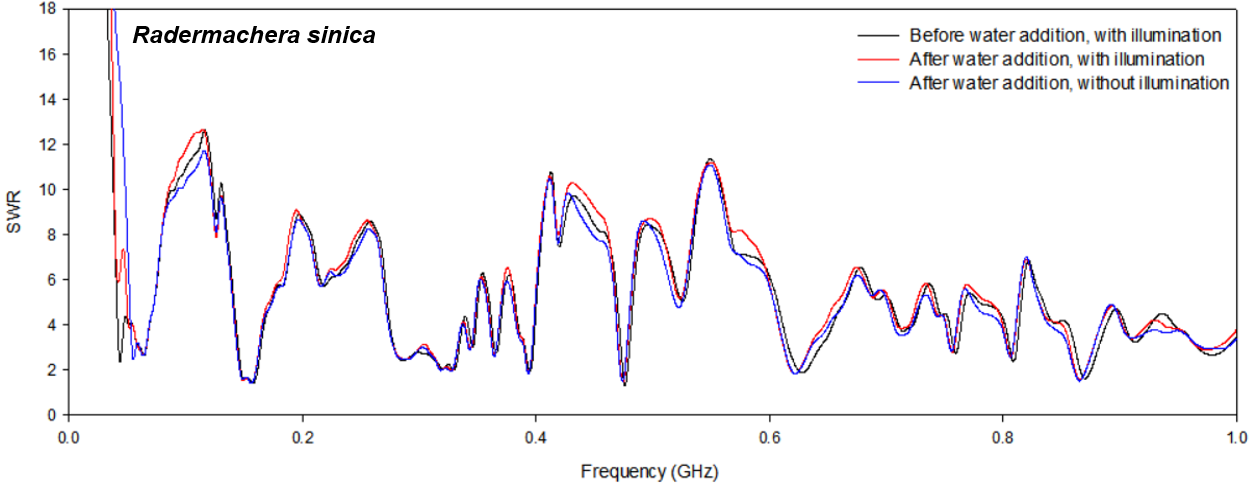


**Additional file 4** Standing wave ratio (SWR) changes for the experimental plant *Radermachera sinica*. SWR measurement of the coil sensor was performed using a network analyzer under various experimental conditions and the frequency was measured at 0.0–1.0 GHz (*p* < 0.05).
